# Serum heme oxygenase‐1 level predicts clinical outcome after acute ischemic stroke

**DOI:** 10.1111/cns.14701

**Published:** 2024-03-27

**Authors:** Huan Wang, Ting Cui, Yaqi Chen, Mingxi Chen, Shihong Zhang, Xinyi Leng, Deren Wang

**Affiliations:** ^1^ Department of Neurology, West China Hospital Sichuan University Chengdu China; ^2^ Center of Cerebrovascular Diseases, West China Hospital Sichuan University Chengdu China; ^3^ Department of Medicine and Therapeutics The Chinese University of Hong Kong Hong Kong SAR China

**Keywords:** biomarker, heme oxygenase‐1, ischemic stroke, prognosis

## Abstract

**Aims:**

The relationship between heme oxygenase‐1 (HO‐1) and human ischemic stroke outcome remains unclear, which was investigated in this study.

**Methods:**

Acute ischemic stroke patients admitted within 24 h were enrolled. Serum HO‐1 levels at baseline were measured via ELISA. Poor 3‐month functional outcome was defined as modified Rankin Scale (mRS) score 3–6. Multivariable‐adjusted binary logistic regression and restricted cubic spline models were employed to examine association between serum HO‐1 and functional outcome. HO‐1's additive prognostic utility was assessed by net reclassification index (NRI) and integrated discrimination improvement (IDI).

**Results:**

Of 194 eligible patients, 79 (40.7%) developed poor functional outcomes at 3‐month follow‐up. The highest quartile of serum HO‐1 was independently associated with a lower risk of poor functional outcome (adjusted OR 0.13, 95% CI 0.04–0.45; *p* = 0.001) compared with the lowest HO‐1 category. The relationship between higher HO‐1 levels and reduced risk of poor functional outcome was linear and dose responsive (*p* = 0.002 for linearity). Incorporating HO‐1 into the analysis with conventional factors significantly improved reclassification for poor functional outcomes (NRI = 41.2%, *p* = 0.004; IDI = 5.0%, *p* = 0.004).

**Conclusions:**

Elevated serum HO‐1 levels at baseline were independently associated with improved 3‐month functional outcomes post‐ischemic stroke. Serum HO‐1 measurement may enhance outcome prediction beyond conventional clinical factors.

## Graphical abstract

Among patients with acute ischemic stroke (AIS), those with higher serum heme oxygenase‐1 (HO‐1) levels at baseline were associated with a lower risk of poor 3‐month functional outcomes. Incorporating HO‐1 with conventional factors improved reclassification for poor functional outcomes. These findings showed the potential of HO‐1 as a prognostic biomarker after stroke.

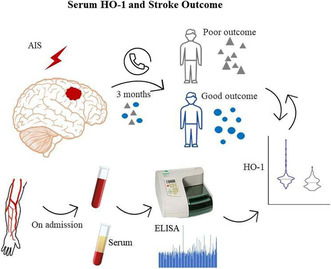

## 1 INTRODUCTION

Accurate prognostication of clinical outcomes subsequent to acute ischemic stroke (AIS) is imperative for informed clinical decision‐making.^1^ Conventional prognostic factors such as age, stroke severity, and hypertension, as well as predictive models predicated on these factors, often lack the requisite precision for guiding clinical decisions.^2^, ^3^, ^4^ The advent of novel blood‐based biomarkers holds promise for augmenting our ability to identify individuals at heightened risk of unfavorable prognosis.^5^

During the occurrence of cerebral ischemia, the production of free radicals and ensuing oxidative stress emerge as a pivotal contributor to causing neuronal damage and subsequent cell demise.^6^, ^7^ Given the compelling association between redox imbalance and ischemic injury, biomarkers affiliated with the endogenous antioxidant system may furnish valuable insights into prognosticating outcomes in individuals afflicted by cerebral infarction.^8^, ^9^ This may open new vistas for therapeutic interventions and clinical decisions. A particularly well‐investigated avenue revolves around the nuclear factor erythroid 2‐related factor 2 (Nrf2) pathway, with heme oxygenase‐1 (HO‐1) serving as a central antioxidant enzyme.^8^, ^10^ Moreover, a clinical study indicates that serum levels of HO‐1 are elevated after hemorrhagic strokes,^11^ and that HO‐1 levels are higher in ischemic strokes than in transient ischemic attacks.^12^

The intracellular enzyme HO‐1 facilitates the degradation of heme into carbon monoxide (CO), biliverdin (BV), and ferrous iron (Fe^2+^), thereby effectively mitigating oxidative stress.^13^, ^14^ Prior research has demonstrated that transgenic mice overexpressing HO‐1 evince significantly diminished infarct volumes in the wake of irreversible occlusion of the middle cerebral artery.^15^ Additionally, therapy with an adenoviral vector augmenting HO‐1 expression engenders reduced infarct volumes and ameliorated neurological deficits in experimental models of brain ischemia, underscoring its neuroprotective potential.^16^ Nevertheless, scant attention has been devoted to exploring the correlation between blood HO‐1 levels and clinical outcomes in stroke patients. Consequently, this study aimed to investigate whether blood HO‐1 levels correlate with poor functional outcomes after acute ischemic stroke.

## 2 METHODS

This study was performed according to the Strengthening the Reporting of Observational Studies in Epidemiology reporting guidelines^17^ and approved by the Ethics Committee on Biomedical Research, West China Hospital of Sichuan University, which conformed to the recommendations of the Declaration of Helsinki. All patients or their relatives provided written informed consent.

### 2.1 Study design and participants

This cohort study was conducted using data from the Chengdu Stroke Registry, an ongoing registry that has prospectively and consecutively enrolled patients with ischemic stroke who were admitted to the Department of Neurology in West China Hospital from 2002. The registry has previously been described in detail.^18^ AIS patients admitted and recorded in the registry, from December 2018 to December 2021, were retrospectively included if they (1) were at least 18 years of age; (2) were admitted to the hospital within 24 h of stroke onset; (3) had a confirmed diagnosis of ischemic stroke based on computed tomography (CT) or magnetic resonance imaging (MRI); (4) had blood samples available for serum HO‐1 measurements; (5) had a modified Rankin Scale (mRS) score of 0–2 prior to stroke; and (6) had completed 3‐month follow‐up. In accordance with the World Health Organization (WHO) criteria, ischemic stroke is defined as acute focal neurological dysfunction caused by single or multiple sites of the brain or retina infarction lasting more than 24 h, or with evidence of acute infarction based on neuroimaging or other technique in the clinically relevant area of the brain.^19^ Patients were excluded if they had (1) major comorbidities or late‐stage diseases, including severe liver disease, heart failure, end‐stage kidney disease, serious infection, malignant tumors, etc.; and (2) concurrent autoimmune diseases.

### 2.2 Data collection

Details of patient demographics, onset‐to‐admission interval, stroke severity on admission, stroke etiology, and vascular risk factors were recorded. The National Institutes of Health Stroke Scale (NIHSS) score was used to assess stroke severity.^20^ Atrial fibrillation (AF) was defined as a history of persistent or paroxysmal AF, confirmed by previous electrocardiogram (ECG) or diagnosed by physician based on an ECG and/or 24‐h ECG monitoring during hospitalization.^21^ Other vascular risk factors included diabetes, hypertension, hyperlipidemia, coronary artery disease, current smoking, alcohol consumption, and history of stroke, which have been described in previous studies.^22^, ^23^ Stroke etiology was classified according to Trial of Org 10172 in Acute Stroke Treatment (TOAST) criteria.^24^ Acute reperfusion therapy, such as intravenous thrombolysis (IVT) and/or endovascular therapy (EVT), was also recorded.

### 2.3 Serum HO‐1 measurement

Peripheral blood samples were collected within 48 h of the patient's arrival at the Emergency Department. Blood samples were centrifuged at clinical laboratories of West China Hospital and then serum samples were immediately frozen at −80°C. Serum concentrations of HO‐1 were detected by laboratory technicians blinded to the clinical data and outcomes of the participants and using enzyme‐linked immunosorbent assay (ELISA) with a commercially available ELISA kit (catalog: MB‐1508A; Jiangsu Meibiao Biotechnology Co., Ltd., Jiangsu, China) in the laboratory of the K J Biotechnology Co., Ltd, Sichuan, China.

### 2.4 Outcome assessment

The mRS was prospectively assessed at 3 months after stroke onset through telephone interviews with patients or family members by well‐trained neurologists. A poor functional outcome at 3 months was defined as an mRS score of 3–6.^25^

### 2.5 Statistical analysis

Categorical variables are expressed as counts with percentages, while continuous data are presented as medians accompanied by their interquartile ranges (IQRs). Participants were grouped according to quartiles of HO‐1 levels. Linear trends in baseline characteristics across quartiles of HO‐1 levels were tested by Cochran–Armitage trend *χ* ^2^ test for categorical variables and generalized linear regression analysis for continuous variables. Univariable and multivariable binary logistic regression models were used to determine the independent associations of the clinical outcomes with HO‐1 quartiles and HO‐1 concentration as a continuous variable. Multivariable ordinal logistic regression was used to assess the association between mRS scores and HO‐1 quartiles. Variables with a potential association with the outcome (*p* < 0.10) from univariate analysis were analyzed via multivariable models. Crude and adjusted odds ratios (ORs) and 95% confidence intervals (CIs) were calculated. To test for linear trend, a term with the median value of each quartile of HO‐1 was entered into the model as a continuous variable.

In addition, the R package rcssci V.0.4.0 was used to generate restricted cubic spline fitted to a logistic regression model in order to provide more precise estimates and explore the shape of the association between serum HO‐1 levels and the clinical outcome.^26^ Three knots for spline were placed at the 10th, 50th, and 90th percentiles of HO‐1, with the 10th percentile serving as a reference point.

To validate the robustness of the association between continuous HO‐1 concentration and poor functional outcome, a subgroup analysis was conducted while adjusting for the aforementioned covariates. Potential interactions between HO‐1 and subgroup variables were probed through likelihood‐ratio tests of models encompassing interaction terms.

Furthermore, the predictive performance of serum HO‐1 as a continuous variable for the clinical outcome was assessed. Predictive models were constructed using either conventional clinical factors alone as the basic model (containing clinical variables with *p* < 0.10 in univariate analyses which were adjusted in the multivariate logistic regression model), or the HO‐1 level combined with these factors. To evaluate the calibration of predictive models, the Hosmer–Lemeshow *χ* ^2^ statistic was used. Net reclassification index (NRI) and integrated discrimination improvement (IDI) were calculated to assess improvements in risk reclassification of model with additive HO‐1 compared with the basic model.^27^

All statistical analyses and graphics were generated using SPSS 24.0 (IBM, New York, USA), R 4.3.0 (the R project for statistical computing), and GraphPad Prism 8.0 (GraphPad Software Inc., San Diego, CA, USA). All *P* values were two‐tailed tests with a statistical significance level of 0.05.

## 3 RESULTS

### 3.1 Baseline characteristics

**FIGURE 1:**
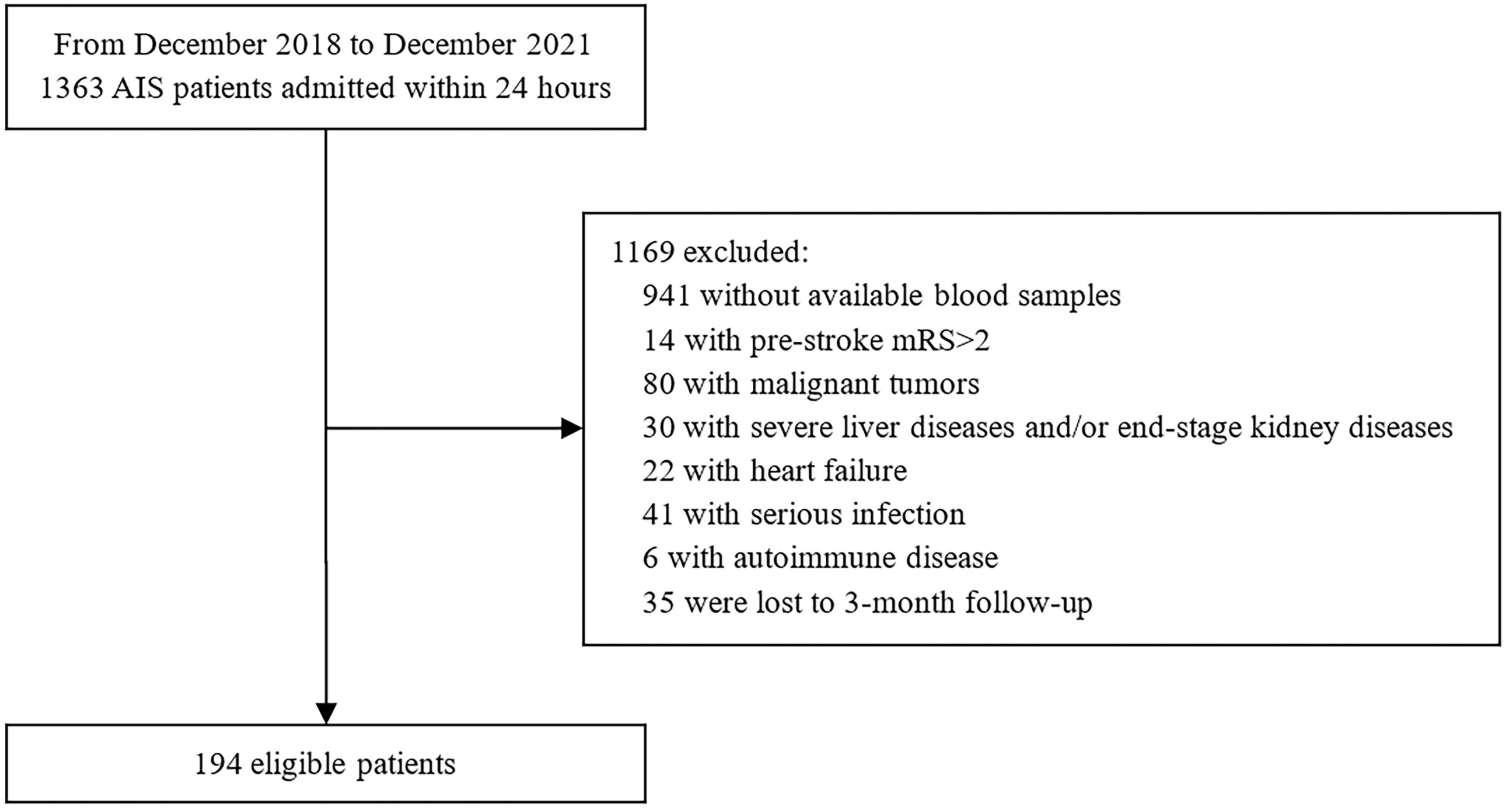
Flow chart for patient screening.

The initial sample consisted of 1363 consecutive AIS patients admitted to our institution within 24 h from December 2018 to December 2021. A total of 194 patients were included in the present study after the exclusion of patients who did not fulfill the selection criteria (Figure 1). There was no significant difference between the baseline characteristics of those included and excluded, except for the stroke onset‐to‐admission interval and alcohol consumption (Table S1). Among 194 included patients, the median age was 66 years, the male proportion was 63.9%, and the median baseline HO‐1 concentration was 28.11 ng/mL (25th and 75th percentiles of 24.05 and 31.03 ng/mL, respectively). In general, higher levels of HO‐1 were associated with younger age, male sex, and lower baseline NIHSS scores (all *p* trends < 0.05; Table 1). Lower HO‐1 concentrations were associated with higher proportions of hypertension, AF, and stroke history, and lower proportions of current smoking and alcohol consumption (all *p* trends < 0.05). More patients with lower HO‐1 concentrations received EVT (*p* trend = 0.009).

**TABLE 1:** Baseline characteristics of participants according to quartiles of HO‐1.

Characteristics	Total	HO‐1 (ng/mL)				*p* trend
		<24.05	24.05–28.11	28.11–31.03	≥31.03	
Number of subjects	194	49	48	49	48	
Demographics						
Age, year	66 (55–72)	70 (65–76)	66 (57–72)	63 (52–72)	59 (51–67)	<0.001^a^
Male	124 (63.9)	23 (46.9)	34 (70.8)	33 (67.3)	34 (70.8)	0.026^a^
Clinical features						
Onset‐to‐admission interval, hour	5.0 (3.0–9.0)	4.0 (2.0–7.3)	4.0 (2.3–9.0)	5.0 (3.5–20.0)	4.8 (3.0–7.8)	0.052
Baseline NIHSS score	8 (3–14)	14 (7–16)	7 (3–13)	6 (2–12)	5 (2–13)	<0.001^a^
Vascular risk factors						
Hypertension	118 (60.8)	34 (69.4)	32 (66.7)	28 (57.1)	24 (50.0)	0.031^a^
Diabetes mellitus	52 (26.8)	17 (34.7)	11 (22.9)	15 (30.6)	9 (18.8)	0.158
Hyperlipidemia	25 (12.9)	6 (12.2)	6 (12.5)	7 (14.3)	6 (12.5)	0.904
Coronary artery disease	22 (11.3)	5 (10.2)	5 (10.4)	8 (16.3)	4 (8.3)	0.982
Atrial fibrillation	59 (30.4)	21 (42.9)	13 (27.1)	14 (28.6)	11 (22.9)	0.048^a^
Current smoking	82 (42.3)	15 (30.6)	19 (39.6)	22 (44.9)	26 (54.2)	0.017^a^
Alcohol consumption	58 (29.9)	9 (18.4)	13 (27.1)	20 (40.8)	16 (33.3)	0.045^a^
Stroke history	19 (9.8)	10 (20.4)	4 (8.3)	3 (6.1)	2 (4.2)	0.007^a^
TOAST classification						
LAA	89 (45.9)	25 (51.0)	24 (50.0)	18 (36.7)	22 (45.8)	0.364
CE	58 (29.9)	18 (36.7)	16 (33.3)	13 (26.5)	11 (22.9)	0.101
SAO	29 (14.9)	3 (6.1)	6 (12.5)	12 (24.5)	8 (16.7)	0.055
SOC	5 (2.6)	2 (4.1)	0 (0.0)	1 (2.0)	2 (4.2)	0.831
SUC	13 (6.7)	1 (2.0)	2 (4.2)	5 (10.2)	5 (10.4)	0.052
Reperfusion therapy						
No	108 (55.7)	23 (46.9)	25 (52.1)	32 (65.3)	28 (58.3)	0.135
IVT	22 (11.3)	4 (8.2)	4 (8.3)	5 (10.2)	9 (18.8)	0.100
EVT	50 (25.8)	18 (36.7)	14 (29.2)	11 (22.4)	7 (14.6)	0.009^a^
IVT and EVT	14 (7.2)	4 (8.2)	5 (10.4)	1 (2.0)	4 (8.3)	0.632

*Note*: Continuous variables are expressed as median (interquartile range). Categorical variables are expressed as frequency (percentage).

Abbreviations: CE, cardioembolism; EVT, endovascular therapy; HO‐1, heme oxygenase‐1; IVT, intravenous thrombolysis; LAA, large artery atherosclerosis; NIHSS, National Institute of Health Stroke Scale; SAO, small artery occlusion; SOC, stroke of other determined cause; SUC, stroke of undetermined cause; TOAST, Trial of Org 10,172 in Acute Stroke Treatment.

^a^ *p* trend < 0.05.

### 3.2 Serum HO‐1 levels and poor functional outcome at 3 months

**FIGURE 2:**
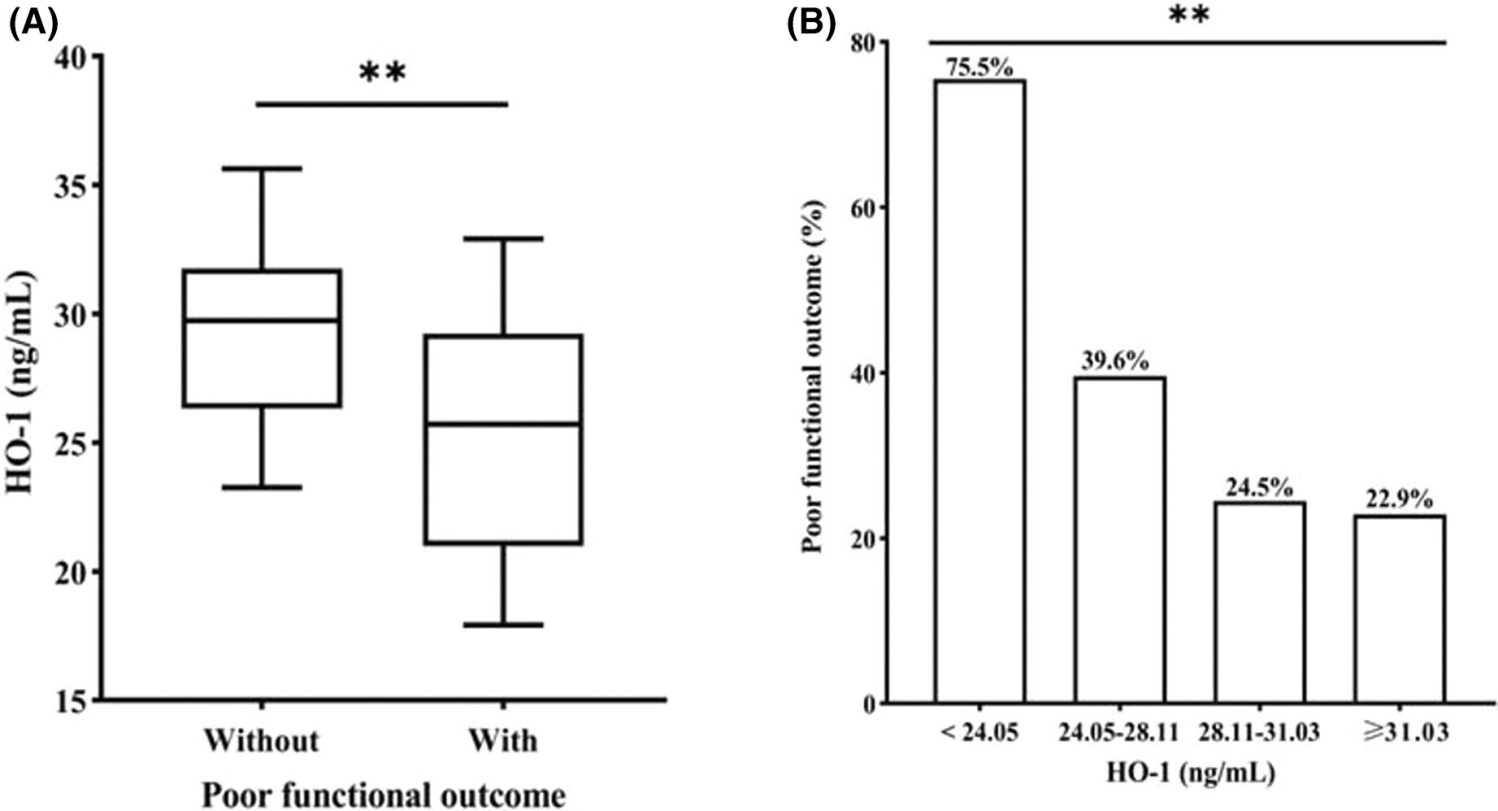
HO‐1 levels according to the clinical outcome. Decreased serum HO‐1 was associated with poor 3‐month functional outcomes after stroke. (A) Lower HO‐1 level in patients with (median 25.72 ng/mL [IQR 21.06–29.41 ng/mL]) versus without poor functional outcome at 3 months (29.74 ng/mL [IQR 26.32–31.76 ng/mL]). Mann–Whitney *U* Test, **p < 0.001. Box plot represents the median and IQR, and whiskers the 10th and 90th percentile. (B) Lower rates of poor functional outcome with higher HO‐1 quartiles. Chi‐squared test, **p < 0.001. HO‐1, heme oxygenase‐1; HT, hemorrhagic transformation; IQR, interquartile range.

**FIGURE 3:**
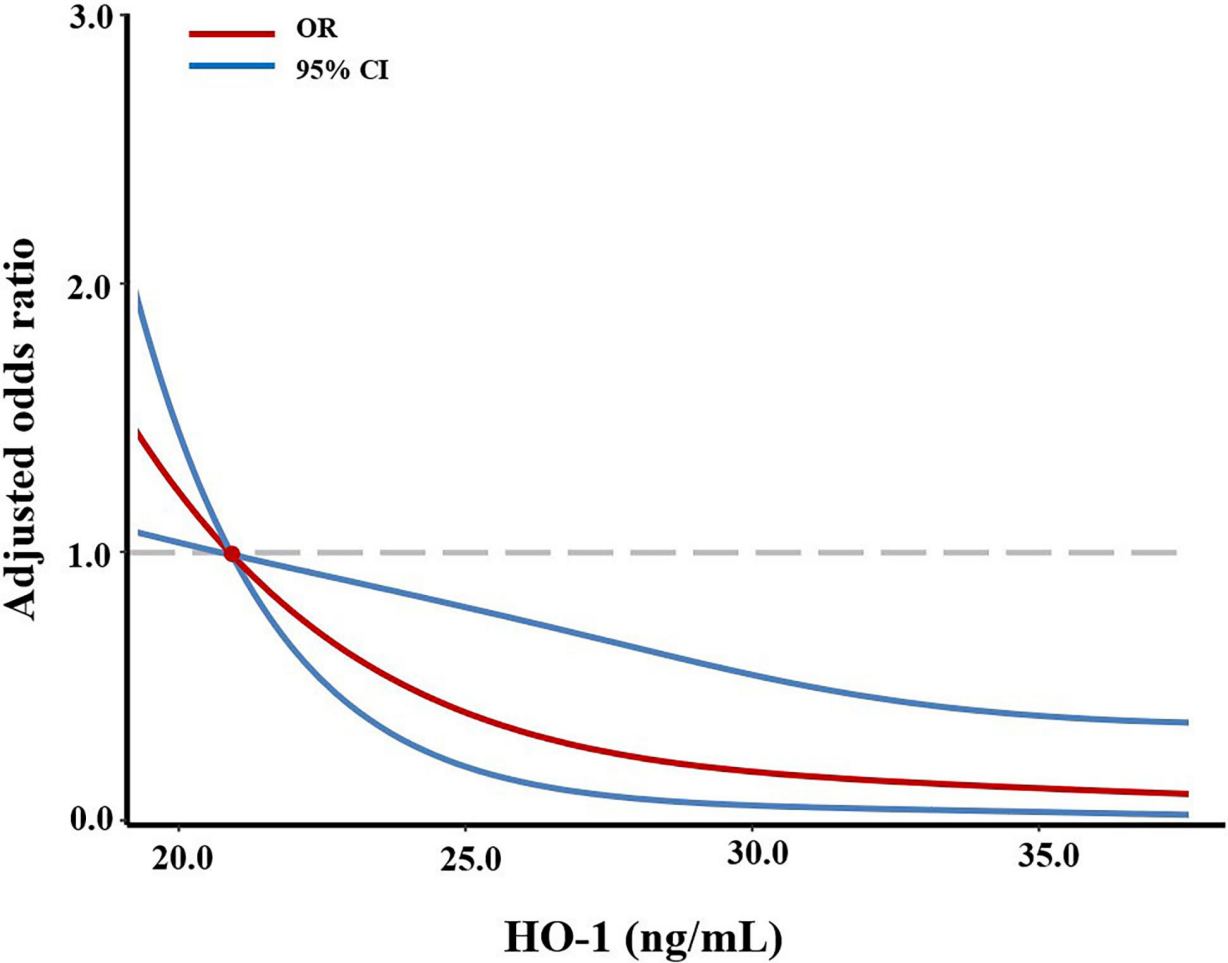
Relationship between HO‐1 levels and clinical outcome of patients with acute ischemic stroke. ORs and 95% CIs derived from restricted cubic spline regression, with knots placed at the 10th, 50th, and 90th percentiles of the distribution of HO‐1 levels. The reference point is the 10th percentile (20.75 ng/mL). A significant inverse linear relationship between serum HO‐1 levels and poor functional outcome at 3 months (*p* = 0.002 for linearity). ORs were adjusted for age, sex, National Institutes of Health Stroke Scale, atrial fibrillation, current smoking, alcohol consumption, and the Trial of ORG 10172 in Acute Stroke Treatment classification and reperfusion therapy. CI, confidence interval; HO‐1, heme oxygenase‐1; OR, odds ratio.

Seventy‐nine patients (40.7%) had poor functional outcomes at 3 months. Serum HO‐1 levels were significantly lower in patients with versus without poor functional outcomes (25.72 vs. 29.74 ng/mL, *p* < 0.001; Figure 2A). A stepwise decrease in the risk of poor functional outcome was observed with higher quartiles of HO‐1 (*p* < 0.001; Figure 2B). Univariable binary logistic regression analysis also showed that a higher HO‐1 level, analyzed either as a continuous or ordinal (in quartiles) variable, was associated with a lower risk of poor functional outcome (all *p* < 0.05; Table S2). The highest quartile (compared with the lowest quartile) of serum HO‐1 was significantly, independently associated with a lower risk of poor functional outcome (adjusted OR 0.13, 95% CI 0.04–0.45; *p* = 0.001; *p*_trend_ < 0.001), after adjusting for age, sex, NIHSS, AF, alcohol consumption, current smoking, TOAST, and reperfusion therapy (Table 2, Figure S1 and Table S3). Moreover, an elevated serum HO‐1 level was also independently associated with a decreased risk of poor functional outcome, when analyzed as a continuous variable (adjusted OR 0.88, 95% CI 0.81–0.95, *p* = 0.001; Table 2). Furthermore, multivariable‐adjusted restricted cubic spline analysis demonstrated an inverse linear relationship between serum HO‐1 levels and poor functional outcomes (*p* = 0.002 for linearity; Figure 3). In addition, there was an inverse dose–response relationship between HO‐1 levels and mRS score at 3 months after ischemic stroke (*p* for trend = 0.004; Figure S2).

**TABLE 2:** The independent association between HO‐1 levels and clinical outcome after stroke adjusted by multivariable binary logistic regression.

	HO‐1 (ng/mL)				*p*_trend_	HO‐1 level as a continuous variable (ng/mL)
	<24.05	24.05–28.11	28.11–31.03	≥31.03		
Median	21.08	26.30	29.74	33.51		
Poor functional outcome (mRS 3–6)						
Events, *n* (%)	37 (75.5)	19 (39.6)	12 (24.5)	11 (22.9)		
Model	Ref	0.39 (0.13–1.18)	0.10 (0.03–0.34)	0.13 (0.04–0.45)		0.88 (0.81–0.95)
*p* value		0.096	<0.001^a^	0.001^a^	<0.001^a^	0.001^a^

*Note*: Model adjusted for age, sex, National Institutes of Health Stroke Scale, atrial fibrillation, current smoking, alcohol consumption, and the Trial of ORG 10172 in Acute Stroke Treatment classification and reperfusion therapy.

Abbreviations: HO‐1, heme oxygenase‐1; Ref, reference.

^a^ *p* value < 0.05.

### 3.3 Subgroup analysis of the association between serum HO‐1 and poor functional outcome

According to the subgroup analyses stratified according to age, sex, hypertension, AF, admission NIHSS score, smoking, alcohol consumption, stroke etiology, and acute reperfusion treatment (with or without IVT and/or EVT), higher serum HO‐1 levels were significantly associated with decreased risk of poor functional outcome in most subgroups (Figure S3). There was no significant interaction between HO‐1 and these stratified factors (all *p* for interaction > 0.05).

### 3.4 Additive prognostic value of serum HO‐1 level for the outcomes

The Hosmer–Lemeshow tests indicated good calibration of the basic prognostic model (containing conventional clinical factors only) and the model with additive HO‐1 level for poor functional outcome (both *p* > 0.05; Table 3). The addition of serum HO‐1 to the basic model notably improved risk reclassification for poor functional outcome (NRI: 41.2% [95% CI 13.5%–68.9%], *p* = 0.004; IDI: 5.0% [95% CI 1.6%–8.3%], *p* = 0.004; Table 3).

**TABLE 3:** Calibration and risk reclassification of models predicting clinical outcome of acute ischemic stroke, with or without HO‐1.

	Calibration		Continuous NRI, %		IDI, %	
	*χ* ^2^	*p* value	Estimate (95% CI)	*p* value	Estimate (95% CI)	*p* value
Poor functional outcome (mRS 3–6)						
Basic model	2.07	0.356	Ref		Ref	
Basic model + HO‐1	0.74	0.691	41.2 (13.5–68.9)	0.004^a^	5.0 (1.6–8.3)	0.004^a^

*Note*: Basic model included age, sex, National Institutes of Health Stroke Scale, atrial fibrillation, current smoking, alcohol consumption, and the Trial of ORG 10172 in Acute Stroke Treatment classification and reperfusion therapy.

Abbreviations: CI, confidence interval; HO‐1, heme oxygenase‐1; IDI, integrated discrimination improvement; mRS, modified Rankin Scale; NRI, net reclassification index; Ref, reference.

^a^ *p* value < 0.05.

## 4 DISCUSSION

Despite extensive basic research demonstrating HO‐1's protective effects against cerebrovascular diseases,^15^, ^28^ data on its impact on clinical outcomes in patients with acute ischemic stroke have been scarce. Therefore, our study explored the relationship between serum HO‐1 levels and the clinical outcome of stroke patients based on data from an ongoing prospective hospital‐based stroke registry. We found higher serum HO‐1 levels independently associated with a lower risk of poor 3‐month functional outcome after an acute ischemic stroke. This association remained consistent across various subgroups, highlighting the robustness of our findings. Notably, the relationship between higher HO‐1 levels and lower risk of poor functional outcome appeared to be linear and dose responsive. Furthermore, incorporating HO‐1 into a predictive model alongside conventional clinical factors significantly improved risk prediction for poor functional outcomes. Our findings shed light on the significant role of HO‐1 in ischemic stroke outcomes and its potential as a prognostic biomarker.

Several previous studies have provided insights into the role of HO‐1 in the context of ischemic stroke. For instance, genetic variations in the HO‐1 gene promoter, leading to increased HO‐1 expression, were associated with a reduced risk of ischemic cerebrovascular events in Austrian patients with normal plasma lipid levels.^29^ Additionally, shorter HO‐1 promoter genotypes were linked to a decreased risk of ischemic stroke, particularly in Chinese individuals with low levels of high‐density lipoprotein cholesterol.^30^ Another study focused on atherosclerotic stroke patients and explored the correlation of HO‐1 gene polymorphisms with clinical prognosis in an Asian cohort, suggesting that patients carrying at least one A allele showed significantly better outcomes compared to those with a TT genotype, potentially attributable to the heightened expression of HO‐1.^31^ Taken together, these findings suggested that HO‐1 level may be related to outcomes after ischemic stroke; however, none of these studies specifically investigated the prognostic role of HO‐1 levels in stroke patients. In addition, while two studies examined the association between HO‐1 levels in the cerebrospinal fluid and functional outcomes in patients with subarachnoid hemorrhage, conflicting findings were found. One study found that higher levels of HO‐1 mRNA were associated with favorable functional outcomes,^32^ whereas in another study, higher levels of HO‐1 protein were associated with more unfavorable outcomes.^33^ Our study attempted to uncover the relationship between serum HO‐1 and long‐term outcomes after stroke, and we found that serum HO‐1 levels are inversely related to poor functional outcomes after 3 months, suggesting the possibility of HO‐1 acting as a neuroprotective target in stroke recovery. Moreover, we demonstrated that the inclusion of serum HO‐1 levels in predictive models could enhance risk prediction for acute ischemic stroke outcomes.

Previous literature suggests that HO‐1 may protect ischemic stroke through its anti‐inflammatory, antioxidant, antiapoptotic, and vasorelaxant effects by degrading the oxidant heme.^8^ Experimental models simulating brain ischemia demonstrated that augmenting HO‐1 expression through gene therapy led to reduced infarct volume and alleviation of neurological impairments.^16^, ^34^ Additionally, HO‐1's downstream metabolites may also exert a protective effect in ischemic stroke. CO exerts a potent anti‐inflammatory effect in diverse diseases, and biliverdin is subsequently transformed into bilirubin,^35^ which can function as an antioxidant. Both of these mechanisms may contribute to the protective role of HO‐1 in cerebral ischemia.^36^, ^37^

There were several limitations to the present study. First, due to the retrospective selection of eligible patients, we had to exclude a considerable number of acute stroke patients without blood samples for HO‐1 measurement. Nevertheless, the large comparability of baseline characteristics between included and excluded patients suggests negligible selection bias. Second, only baseline serum HO‐1 levels were measured, while early complications after an index stroke or other conditions may affect the HO‐1 levels; hence, serial measurements could potentially provide more comprehensive prognostic information. Future studies are needed to investigate the associations between dynamic variations in serum HO‐1 levels and the prognosis of ischemic stroke. Third, the possibility of residual confounding factors remains, as treatments beyond acute reperfusion therapies were not considered in our multivariate analyses. Finally, our study cohort comprised solely Chinese patients, warranting caution when generalizing the findings to other populations. Therefore, further large‐scale prospective studies conducted among different populations are needed to replicate our findings.

## 5 CONCLUSION

Our study highlights the independent association between higher serum HO‐1 levels on baseline and a reduced risk of poor functional outcome at 3 months following ischemic stroke. Serum HO‐1 levels demonstrate potential as a prognostic biomarker for acute ischemic stroke outcome, offering additive prognostic value when integrated with conventional clinical factors. To validate and extend our findings, further prospective studies with larger and more diverse samples, serial assessments of HO‐1 levels post‐stroke, and examination in different populations are warranted.

## FUNDING INFORMATION

This work was supported by the Sichuan Science and Technology Program (No. 2023YFH0096), the National Key Research and Development Program of China (No. 2018YFC1705006), the National Natural Science Foundation of China (Nos. 82271331 and 81974208), and the 1·3·5 project for disciplines of excellence‐Clinical Research Incubation Project of West China Hospital (No. 2018HXFH041).

## CONFLICT OF INTEREST STATEMENT

None.

## Supporting information


Appendix S1.

## Data Availability

Further anonymized data can be made available upon reasonable request from a qualified investigator to the corresponding author.
