# Supplementary material for: Serum heme oxygenase‐1 level predicts clinical outcome after acute ischemic stroke
Source: CNS Neurosci Ther. 2024 Mar 27;30(3):e14701. doi: 10.1111/cns.14701 (PMC10973699; doi:10.1111/cns.14701)
Supplement: Supplementary file 1 — Appendix S1. [file CNS-30-e14701-s001.docx]

**Supplemental Material**

Supplemental Tables and supporting information (Supplemental Table 1-3)

Supplemental Figures and figure legends (Supplemental Figure 1-3)

**Supplementary Table 1. Characteristics of patients admitted within 24 hours from December 2018 to December 2021, excluded or included in the current study.**

| **Characteristics** | **Included (n=194)** | **Excluded (n=1169)** | ***P* value** |
| --- | --- | --- | --- |
| **Demographics** |  |  |  |
| **Age, y** | 66 (55-72) | 67 (56-74) | 0.057 |
| **Male** | 124 (63.9) | 722 (61.8) | 0.567 |
| **Clinical features** |  |  |  |
| **Onset-to-admission interval, h** | 5.0 (3.0-9.0) | 5.0 (3.0-14.0) | 0.003* |
| **Baseline National Institute of Health Stroke Scale score** | 8 (3-14) | 9 (3-15) | 0.274 |
| **Vascular risk factors** |  |  |  |
| **Hypertension** | 118 (60.8) | 701 (60.0) | 0.821 |
| **Diabetes mellitus** | 52 (26.8) | 345 (29.5) | 0.442 |
| **Hyperlipidemia** | 25 (12.9) | 164 (14.0) | 0.670 |
| **Coronary artery disease** | 22 (11.3) | 119 (10.2) | 0.623 |
| **Atrial fibrillation** | 59 (30.4) | 352 (30.1) | 0.933 |
| **Current smoking** | 82 (42.3) | 410 (35.1) | 0.053 |
| **Alcohol consumption** | 58 (29.9) | 236 (20.2) | 0.002* |
| **Stroke history** | 19 (9.8) | 126 (10.8) | 0.680 |

Continuous variables are expressed as median (interquartile range). Categorical variables are expressed as frequency (percentage).

**P* value<0.05.

**Supplementary Table 2. Associations between baseline characteristics and poor functional outcome after stroke in univariable binary logistic regression.**

| **Variables** | **Unadjusted OR (95% CI)** | ***P* value** |
| --- | --- | --- |
| **Age** | 1.06 (1.03-1.09) | <0.001* |
| **Sex** | 0.60 (0.33-1.09) | 0.096^#^ |
| **Onset-to-admission interval** | 1.01 (0.97-1.05) | 0.673 |
| **Baseline National Institute of Health Stroke Scale score** | 1.23 (1.16-1.31) | <0.001* |
| **Hypertension** | 1.09 (0.61-1.96) | 0.776 |
| **Diabetes mellitus** | 1.09 (0.57-2.08) | 0.786 |
| **Hyperlipidemia** | 0.65 (0.27-1.59) | 0.344 |
| **Coronary artery disease** | 1.24 (0.51-3.04) | 0.632 |
| **Atrial fibrillation** | 3.35 (1.77-6.33) | <0.001* |
| **Current smoking** | 0.52 (0.29-0.94) | 0.030* |
| **Alcohol consumption** | 0.50 (0.26-0.96) | 0.036* |
| **Stroke history** | 2.16 (0.83-5.65) | 0.115 |
| **Trial of Org 10172 in Acute Stroke Treatment classification** |  |  |
| **Large artery atherosclerosis** | Ref |  |
| **Cardioembolism** | 1.82 (0.93-3.55) | 0.081^#^ |
| **Small artery occlusion** | 0.10 (0.02-0.42) | 0.002* |
| **Stroke of other determined cause** | 0.86 (0.14-5.37) | 0.867 |
| **Stroke of undetermined cause** | 0.23 (0.05-1.11) | 0.068^#^ |
| **Reperfusion therapy** |  |  |
| **No** | Ref |  |
| **Intravenous thrombolysis** | 0.86 (0.32-2.29) | 0.762 |
| **Endovascular therapy** | 2.54 (1.28-5.05) | 0.008* |
| **Intravenous thrombolysis and Endovascular therapy** | 1.02 (0.32-3.27) | 0.969 |
| **HO-1 level (as a continuous variable)** | 0.86 (0.80-0.92) | <0.001* |
| **HO-1** |  |  |
| **Quartile 1** | Ref |  |
| **Quartile 2** | 0.21 (0.09-0.51) | <0.001* |
| **Quartile 3** | 0.11 (0.04-0.26) | <0.001* |
| **Quartile 4** | 0.10 (0.04-0.25) | <0.001* |

Abbreviations: HO-1, Heme Oxygenase-1; OR, odds ratio; CI, confidence interval; Ref, reference.

**P* value<0.05.

^#^*P* value<0.10.

**Supplementary Table 3. The independent associations between selected variables and poor functional outcome after stroke in multivariable binary logistic regression.**

| **Variables** | **Adjusted OR (95% CI)** | ***P* value** |
| --- | --- | --- |
| **Age** | 1.04 (1.00-1.08) | 0.060 |
| **Sex** | 1.35 (0.47-3.92) | 0.577 |
| **Baseline National Institute of Health Stroke Scale score** | 1.26 (1.15-1.38) | <0.001* |
| **Atrial fibrillation** | 4.50 (0.52-39.00) | 0.173 |
| **Current smoking** | 0.59 (0.18-1.94) | 0.383 |
| **Alcohol consumption** | 0.92 (0.27-3.19) | 0.898 |
| **Trial of Org 10172 in Acute Stroke Treatment classification** |  |  |
| **Large artery atherosclerosis** | Ref |  |
| **Cardioembolism** | 0.33 (0.04-2.76) | 0.304 |
| **Small artery occlusion** | 0.34 (0.06-1.81) | 0.205 |
| **Stroke of other determined cause** | 0.54 (0.04-7.51) | 0.642 |
| **Stroke of undetermined cause** | 0.86 (0.15-5.12) | 0.869 |
| **Reperfusion therapy** |  |  |
| **No** | Ref |  |
| **Intravenous thrombolysis** | 0.89 (0.22-3.58) | 0.869 |
| **Endovascular therapy** | 0.35 (0.11-1.10) | 0.073 |
| **Intravenous thrombolysis and Endovascular therapy** | 0.07 (0.01-0.45) | 0.005* |
| **HO-1** |  |  |
| **Quartile 1** | Ref |  |
| **Quartile 2** | 0.39 (0.13-1.18) | 0.096 |
| **Quartile 3** | 0.10 (0.03-0.34) | <0.001* |
| **Quartile 4** | 0.13 (0.04-0.45) | 0.001* |

Abbreviations: HO-1, Heme Oxygenase-1; OR, odds ratio; CI, confidence interval; Ref, reference.

Model included age, sex, National Institutes of Health Stroke Scale, atrial fibrillation, current smoking, alcohol consumption, the Trial of ORG 10172 in Acute Stroke Treatment classification, reperfusion therapy and HO-1 levels.

**P* value<0.05.


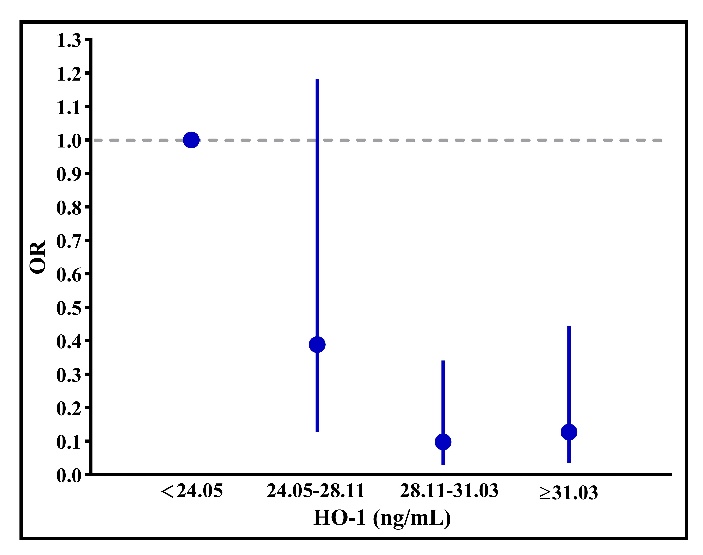


**Supplementary Figure 1. ORs and 95% CIs for HO-1 categories.** The presented ORs are based on the binary logistic regression analysis to evaluate the association between HO-1 levels and clinical outcome (from Table 2). Abbreviations: HO-1, Heme Oxygenase-1; OR, odds ratio; CI, confidence interval.


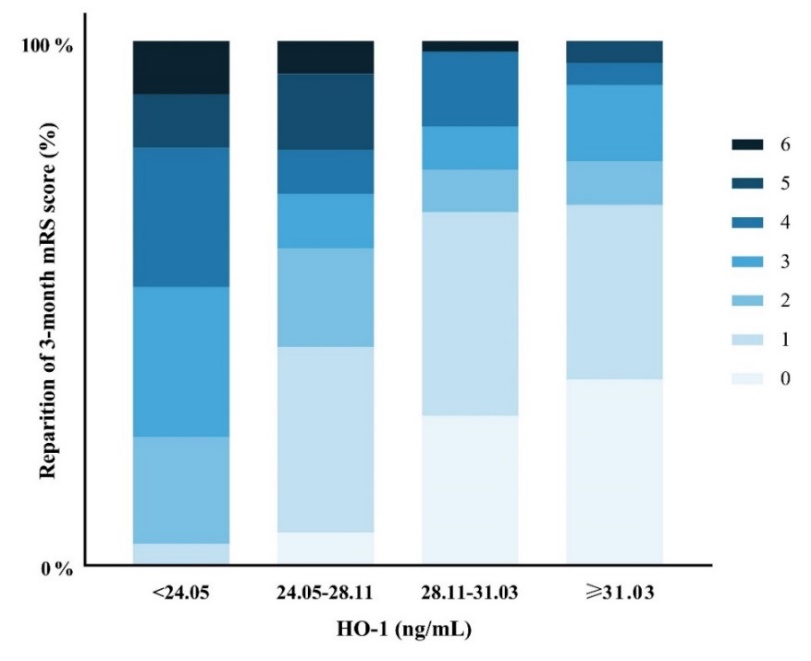


**Supplementary Figure 2. Distribution of 3-month mRS according to HO-1 quartiles.** More patients with higher 3-month mRS in those with lower levels of HO-1 in quartiles. Multivariable-adjusted odds ratio of ordinal logistic regression analysis was 0.25 (95% CI 0.07-0.91) for patients with the HO-1 levels in the highest versus lowest quartile (*P* for trend=0.004). Variables adjusted in the multivariable model included age, sex, National Institutes of Health Stroke Scale, atrial fibrillation, current smoking, alcohol consumption, the Trial of ORG 10172 in Acute Stroke Treatment classification and reperfusion therapy. Abbreviations: HO-1, Heme Oxygenase-1; mRS, modified Rankin Scale.


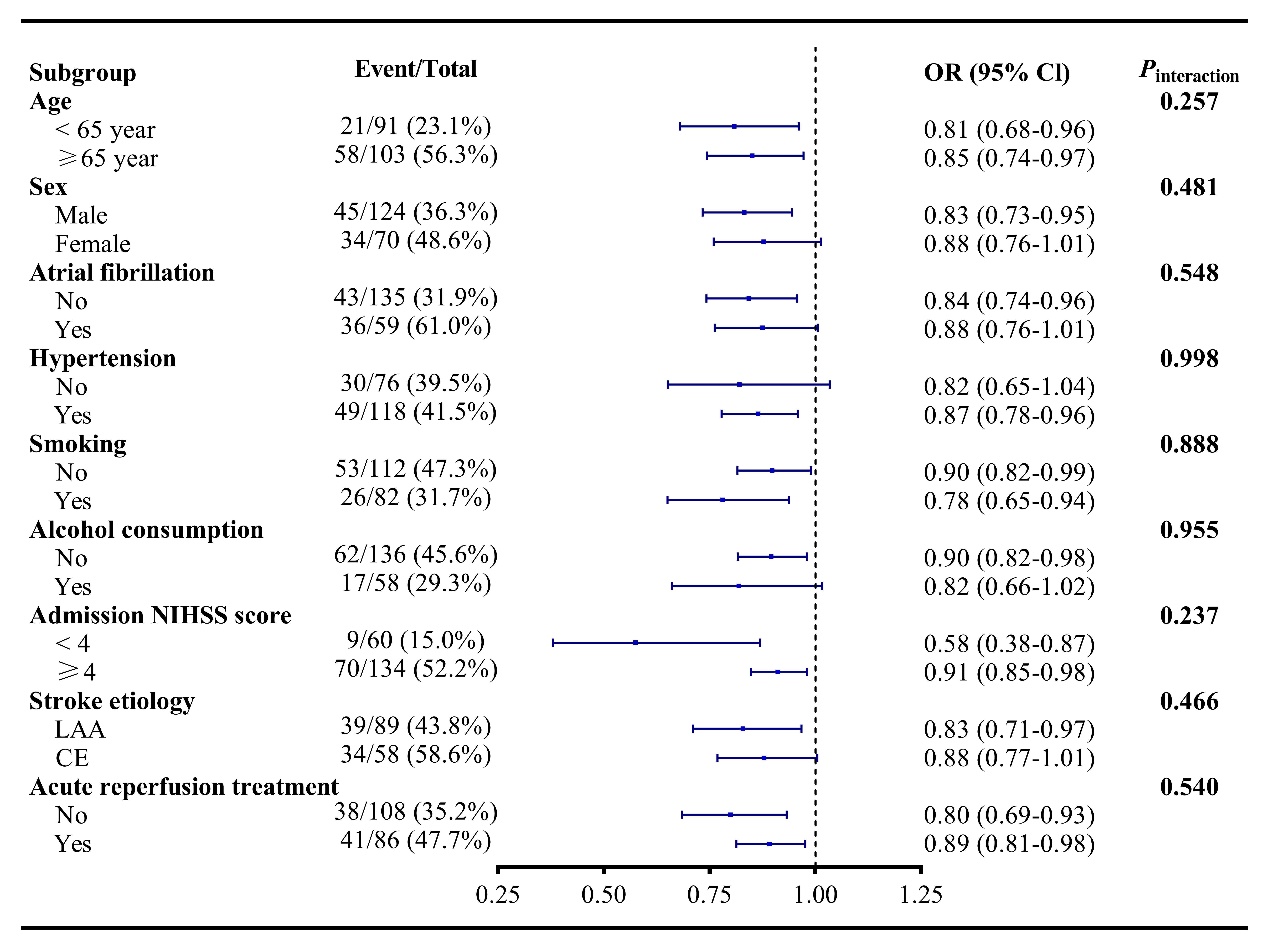


**Supplementary Figure 3. Subgroup analysis of association between HO-1 level (as a continuous variable) and poor functional outcome in acute ischemic stroke patients.** The presented ORs and 95% CIs are based on binary logistic regression analyses with adjustment for age, sex, National Institutes of Health Stroke Scale, atrial fibrillation, current smoking, alcohol consumption, the Trial of ORG 10172 in Acute Stroke Treatment classification and reperfusion therapy, except for the stratified variable. Abbreviations: HO-1, Heme Oxygenase-1; OR, odds ratio; CI, Confidence interval.
